# Schistosomiasis Screening of Travelers to Corsica, France

**DOI:** 10.3201/eid2201.151590

**Published:** 2016-01

**Authors:** Anna Beltrame, Lorenzo Zammarchi, Gianluca Zuglian, Federico Gobbi, Andrea Angheben, Valentina Marchese, Monica Degani, Antonia Mantella, Leila Bianchi, Carlotta Montagnani, Luisa Galli, Matteo Bassetti, Alessandro Bartoloni, Zeno Bisoffi

**Affiliations:** Sacro Cuore Hospital, Negrar, Italy (A. Beltrame, F. Gobbi, A. Angheben, V. Marchese, M. Degani, Z. Bisoffi);; Santa Maria Misericordia University Hospital of Udine, Udine, Italy (G. Zuglian, M. Bassetti);; University of Florence School of Medicine, Florence, Italy (L. Zammarchi, A. Mantella, A. Bartoloni);; Anna Meyer Children’s University Hospital, Florence, Italy (L. Bianchi, C. Montagnani, L. Galli)

**Keywords:** schistosomiasis, screening, travelers, exposure, surveillance, Schistosoma, haematobium, mansoni, parasites, trematode, snail, intermediate host, urinary schistosomiasis, Bulinus truncatus snail, One Health, freshwater rivers, SCHISTO II WB IgG test, Western blot, Cavu River, Corsica, France, Italy, Europe

**In Response:** Regarding the comments by Berry et al. (*1*) on our previously published letter, we acknowledge that, in strict parasitological terms, confirmation of the diagnosis of urogenital schistosomiasis requires the identification of eggs by microscopic examination of urine. Nevertheless, we aimed at an operational case definition, providing criteria for identifying cases most likely to be true infections. We should not forget that microscopy has an unacceptably low sensitivity (*2*). We should also consider that currently available serologic tools are hampered by both a poor sensitivity and a poor specificity for *Schistosoma haematobium* (*3*). Regarding immunoblot, Berry et al. are correct in saying that there is not yet any formally published evidence of its accuracy for *S. haematobium* and that the high specificity declared, close to 100%, is based on data provided by the manufacturer. A formal study on the accuracy of this test is underway at the Centre for Tropical Diseases of Sacro Cuore Hospital. This assay has been less extensively assessed than that in which purified *S. mansoni* antigen is used, as described previously, which has shown very high accuracy (*4*). However, Western blot is already accepted as a diagnostic standard for the identification of other infectious diseases, including parasitic infections such as cysticercosis (for which, indeed, the direct parasitological confirmation is often impossible), and has become the test of choice for the latter (*5*). 

Moreover, the population in our study was composed of persons not exposed to other parasites. Therefore, cross-reactions with other helminths would be extremely unlikely.

In conclusion, although we recognize that, by a strictly semantic definition, the term “confirmed” should be reserved for cases for which there is a parasitological proof, in operational terms, we could not rely on a direct test that has such a poor sensitivity in this particular patient population. Had we done so, we would have found a subestimated, and therefore totally incorrect, picture of the true prevalence, leading to inappropriate conclusions and actions (or lack thereof).
